# Resveratrol induces mitochondria-mediated, caspase-independent apoptosis in murine prostate cancer cells

**DOI:** 10.18632/oncotarget.14947

**Published:** 2017-02-01

**Authors:** Sanjay Kumar, Erdal Eroglu, James A. Stokes, Karyn Scissum-Gunn, Sabita N. Saldanha, Udai P. Singh, Upender Manne, Selvarangan Ponnazhagan, Manoj K. Mishra

**Affiliations:** ^1^ Cancer Biology Research and Training, Department of Biological Sciences, Alabama State University, Montgomery, AL, USA; ^2^ Faculty of Engineering, Bioengineering Department, Celal Bayar University, Muradiye, Manisa, Turkey; ^3^ Department of Pathology, Microbiology and Immunology, University of South Carolina, Columbia, SC, USA; ^4^ Department of Pathology, School of Medicine, University of Alabama at Birmingham, Birmingham, AL, USA

**Keywords:** apoptosis, mitochondria, prostate cancer cells, resveratrol

## Abstract

Found in the skins of red fruits, including grapes, resveratrol (RES) is a polyphenolic compound with cancer chemopreventive activity. Because of this activity, it has gained interest for scientific investigations. RES inhibits tumor growth and progression by targeting mitochondria-dependent or -independent pathways. However, further investigations are needed to explore the underlying mechanisms.

The present study is focused on examining the role of RES-induced, mitochondria-mediated, caspase-independent apoptosis of prostate cancer cells, namely transgenic adenocarcinoma of mouse prostate (TRAMP) cells. These cells were exposed to RES for various times, and cell killing, cell morphology, mitochondrial membrane potential (Δψm), expression of Bax and Bcl2 proteins, the role of caspase-3, and DNA fragmentation were analyzed.

TRAMP cells exposed to RES showed decreased cell viability, altered cell morphology, and disrupted Δψm, which led to aberrant expression of Bax and Bcl2 proteins. Furthermore, since the caspase-3 inhibitor, z-VAD-fmk (benzyloxycarbonyl-valine-alanine-aspartic acid-fluoromethyl ketone), had no appreciable impact on RES-induced cell killing, the killing was evidently caspase-independent. In addition, RES treatment of TRAMP-C1, TRAMP-C2, and TRAMP-C3 cells caused an appreciable breakage of genomic DNA into low-molecular-weight fragments.

These findings show that, in inhibition of proliferation of TRAMP cells, RES induces mitochondria-mediated, caspase-independent apoptosis. Therefore, RES may be utilized as a therapeutic agent to control the proliferation and growth of cancer cells.

## INTRODUCTION

In Western populations, prostate cancer is the second leading cause of death (after heart disease) in men older than 65 years of age [1–6]. It arises through the change of pre-neoplastic lesions into adenocarcinomas, and thereafter progresses to metastatic disease [6–9]. Recent advances have found genetic alterations that enhance the probability of prostate cancer development [10, 11]. To limit the growth of prostate cancer, high doses of chemotherapeutic drugs and high-frequency radiation have been used, but the limited efficacy and side effects of these treatments raise a concern [12, 13]. Therefore, it is desirable to find anti-cancer agents that are non-toxic and effective in inducing apoptosis in cancer cells. Previous studies have demonstrated that treatment with anti-androgens is beneficial in the early stages of prostate cancer development, suggesting that their growth may be dependent on androgens [14–17]. In contrast, androgen-independent prostate cancer cells do not undergo apoptosis [14–17]. In view of these results, advanced therapies that target the proliferation of both androgen-dependent and -independent prostate cancer cells are needed.

Compounds naturally occurring and present in the human diet are generally nontoxic, and some have beneficial effects on human health. Among these dietary components, resveratrol (RES), a polyphenol found in the skin of red fruits, exhibits anti-cancer, anti-proliferative, anti-inflammatory, and anti-oxidative effects [18, 19]. The anti-cancer properties of RES are facilitated through various changes in apoptotic signaling, metabolic pathways, and other signaling pathways that regulate apoptosis, cell cycle progression, inflammation, proliferation, metastasis, and angiogenesis [18, 20]. Furthermore, cells exposed to RES show inhibition of PI3K/AKT signaling, which stimulates apoptosis [21–29]. RES induces the death receptor (TRAIL/DR4 and TRAIL-R2/DR5)-mediated apoptosis [7, 30–39], and it is involved in mitochondrial-mediated apoptosis [40–45]. Mitochondria are the primary provider of ATP in most mammalian cells, they regulate both necrotic and apoptotic cell death pathways [46]. Thus, apoptosis is considered to be the most likely mechanism adopted by cells after activation of death signals. Apoptosis may be triggered intrinsically or extrinsically, depending on the type of apoptotic signals [40, 41, 43–45].

In human lung adenocarcinoma cells, RES activates the intrinsic apoptotic pathway by inducing release of apoptosis-inducing factor (AIF) [47]. Intrinsic signals of apoptosis function mainly through mitochondria [48]. In healthy cells, the outer mitochondrial membrane expresses the B-cell lymphoma-2 (Bcl-2) family of proteins, which controls the release of pro-apoptotic factors from the inner-membrane space in mitochondria [49–52]. In response to internal damage to the cells, a Bcl-2 associated protein, Bax, migrates to the mitochondrial membrane and inhibits the action of Bcl-2, causing damage to the mitochondrial membrane that in turn releases cytochrome-c [49–52]. Cytochrome-c binds with the apoptotic protease activator factor-1 (Apaf-1) and forms a multimeric protein structure called the “apoptosome.” The apoptosome activates caspase-9, which triggers the activation of caspase-3 and caspase-7 [53–59]. Their activation initiates proteolytic activity that leads to cell death [53–59]. The extrinsic pathway of apoptosis, however, is triggered by external signals that stimulate death receptors, such as ligands Fas-L and TNF-α (tumor necrosis factor-α), which activate caspase-8 [60]. This activated molecule initiates a cascade of caspase activity, which facilitates cell death [60].

RES shows anti-cancer, anti-proliferative, anti-inflammatory, and anti-oxidative properties, which are involved in the mitochondrial pathway of apoptosis [18, 20, 40–44, 61, 62]. However, how RES-induced, mitochondria-mediated, caspase-independent apoptosis operates in controlling the progression of tumor cells is not clear.

In the present effort, we examined the effects of RES on mitochondria-mediated, caspase-independent apoptosis in transgenic adenocarcinoma of mouse prostate (TRAMP-C1, TRAMP-C2, and TRAMP-C3) cells. TRAMP cells exposed to RES showed, in a time- and dose-dependent manner, increased cell killing and altered cell morphology. Furthermore, RES treatment resulted in disrupted mitochondrial membrane potential (Δψm), which triggered disproportionate expression of Bax and Bcl2 proteins. In addition, RES treatment did not induce marked fragmentation of DNA into low-molecular-weight segments. As determined by exposure of cells to the caspase-3 inhibitor, z-VAD-fmk, caspase-3 was not involved in RES-mediated cell killing. Thus, these findings indicate that RES induces mitochondria-mediated, caspase-independent apoptosis and delays proliferation of prostate cancer cells. Therefore, RES may be an agent for treatment of prostate cancer.

## RESULTS

### RES kills tumor cells

**Figure 1:**
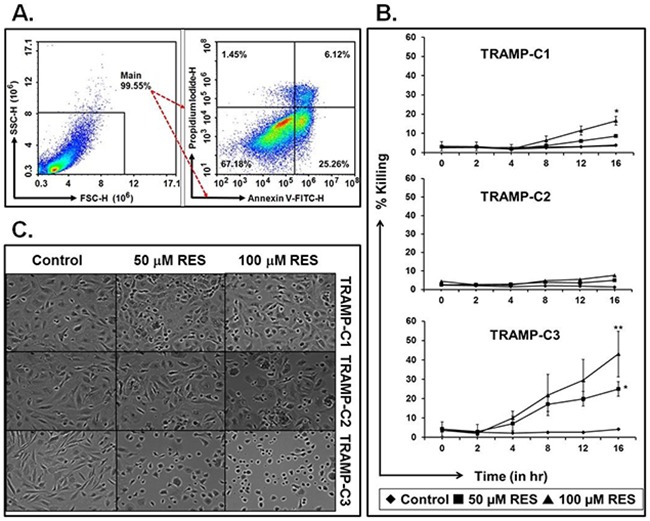
RES kills TRAMP cells in a dose- and time-dependent manner TRAMP cells were treated with RES (50 μM or 100 μM), and cell killing and cell morphology were examined. **A**. Representative figure of gating strategies to study percent cell killing after RES treatment using a flow cytometer. **B**. Mean average values with ±SEM of cell death at 0, 2, 4, 8, 12, and 16 hours. **C**. Morphological changes in cells due to RES treatment (* indicates p<0.05 and ** indicates p<0.01).

To test the effect of RES on TRAMP cells, a cell-killing assay was performed. First, we determined the optimal time and concentration of RES needed to kill TRAMP cells. Annexin V-FITC^+^ and double positive (FITC^+^, PI^+^) cells showed sign of early and late apoptosis respectively; however, PI+ cells considered as dead cells as shown in representative Figure 1A. We established that 16 hours was the optimal time for maximum killing (Figure 1B). These experiments were repeated 5 times independently in triplicates. Data are represented as mean values ±SEM (Standard Error of the Mean) in Figure 1B. We also conducted experiments at 24 hours and 48 hours but did not find any significant difference in RES-mediated cell killing (data not shown). Cells incubated with 50 μM or 100 μM of RES showed a concentration-dependent increase in the percent of cells killed (Figure 1B). Further analysis revealed that RES (100 μM) treatment resulted in a significantly greater (*P<0.001) killing of TRAMP-C3 cells (43±5%) as compared to TRAMP-C1 (21±5%) and TRAMP-C2 (6±5%) cells (Figure 1B). In addition, TRAMP cells were incubated with RES in the presence or absence of Nec-1, a necroptosis blocker, to confirm whether RES-mediates apoptosis or necroptosis. We found that RES exhibited a similar pattern of cell killing in the presence or absence of Nec-1 (Supplementary Figure 1A; TRAMP-C1, Supplementary Figure 1B; TRAMP-C2, and Supplementary Figure 1C; TRAMP-C3).

### RES treatment alters cell morphology

To evaluate the impact of RES on cell morphology, phase contrast microscopy was conducted. Cells were treated with 100 μM of RES for 16h exhibited altered cell morphology in a concentration-dependent manner (Figure 1C). Furthermore, cells exposed to 100 μM of RES showed more prominent morphological alterations in TRAMP as compared to cells treated with 50 μM of RES (Figure 1C). Additionally, TRAMP-C3 cells were more sensitive to 100 μM of RES as compared to TRAMP-C1 and TRAMP-C2 cells (Figure 1C). TRAMP-C3 cells showed more oval shapes as compared to TRAMP-C1 and TRAMP-C2 cells, suggesting loss of adherence and loss of cell-to-cell contact.

### RES induces mitochondrial membrane potential

**Figure 2:**
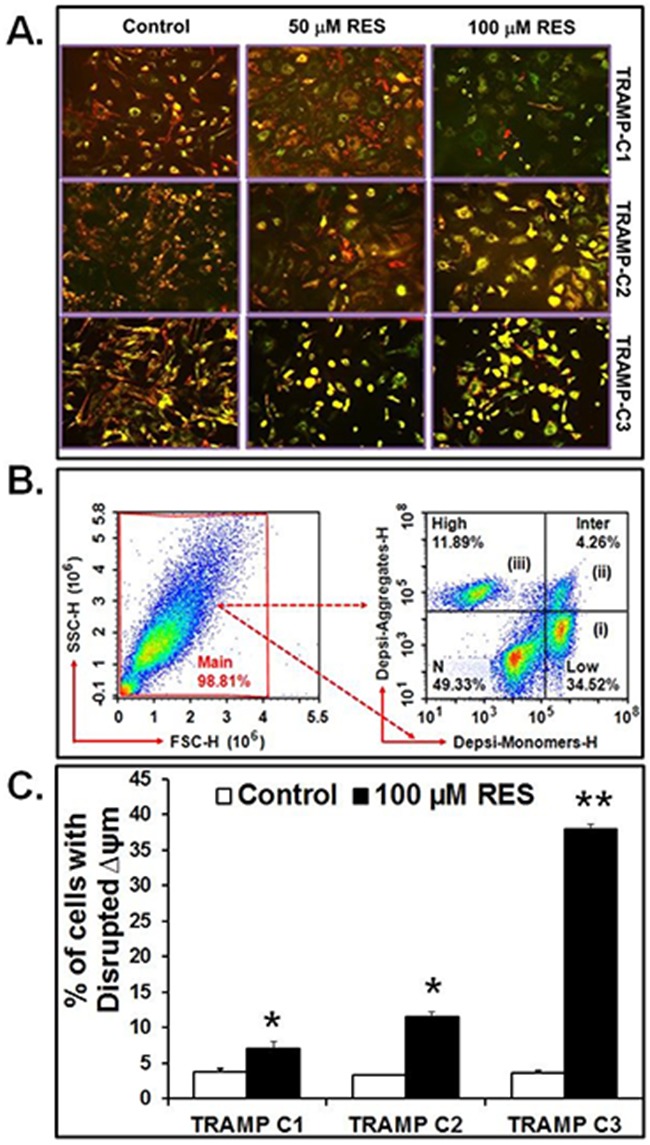
RES disrupts Δψm Cells were treated with 100 μM of RES for 16 hours, and Δψm was examined by using DePsipher dye. **A**. Fluorescence microscopy of TRAMP cells. **B**. Representative gating of cells. (i) cells with disrupted (low) Δψm are indicated as DePsipher-monomers (34.52%), (ii) cells with intermediate Δψm (4.26%), and (iii) cell with high Δψm indicated as DePsipher-aggregates (11.89%). **C**. Percent of cells with disrupted Δψm. Data represented as mean values ± SEM (* indicates p<0.05 and ** indicates p<0.01).

To examine the effect of RES on mitochondria, the Δψm was measured using fluorescence microscopy and flow cytometry. TRAMP cells treated with RES (50 or 100 μM) showed disrupted Δψm as compared to appropriate control (Figure 2A). Observed under a fluorescence microscope, DePsipher-stained TRAMP cells exhibited a distinct fluorescence color: red, green, or an overlap of green and red that results in orange/yellow. Cells with red fluorescence were considered to be healthy and normal; cells with green fluorescence were considered to have disrupted Δψm, indicating apoptosis. Cells with orange/yellow fluorescence were considered to have collapsed mitochondria. Most of the treated TRAMP cells showed green and orange/yellowish color, indicating that these cells had disrupted Δψm (Figure 2A); however, TRAMP-C3 cells showed significantly disrupted Δψm as compared to TRAMP-C1 and TRAMP-C2 cells (Figure 2A).

Further, the Δψm was validated by flow cytometric analysis of TRAMP cells treated with 100 μM RES as demonstrated in representative Figure 2B. In this figure, three cell populations were evident: (i) TRAMP cells showing only green fluorescence (FL1: 488/530 nm) corresponding to those with disrupted (low) Δψm following apoptosis as compared to control cells. (ii) Cells with different intensities of green and red or yellow/orange were also considered to have intermediate disrupted Δψm. (iii) Cells emitting red fluorescence (FL2: 488/585nm) were considered to demonstrate high Δψm. Further analysis revealed that, with exposure to 100 μM RES, the percentages of TRAMP-C3 cells showing green fluorescence, that is, with disrupted Δψm, were higher relative to TRAMP-C1 and TRAMP-C2 cells (Figure 2C). This experiment was performed 5 times independently in triplicates; the sum of all experimental data (± SEM) is shown in the histogram Figure 2C (*p<0.05 and **p<0.01).

### RES modulates the expression of Bax and Bcl2

**Figure 3:**
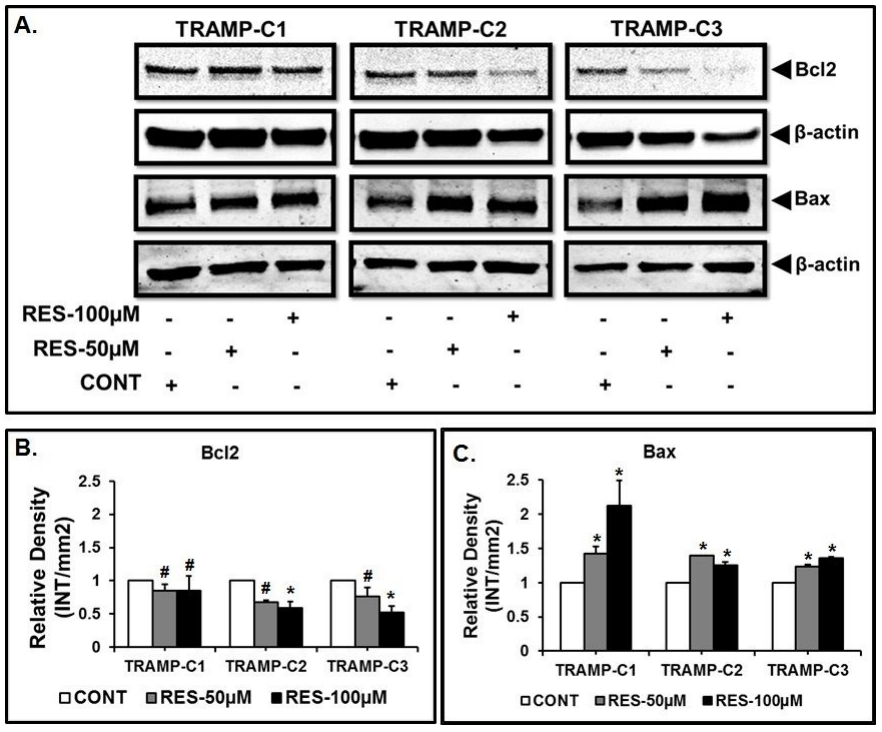
RES modulates the expression of Bax and Bcl2 Results indicated altered expression of Bax and Bcl2 in TRAMP cells after Res treatment. **A**. Representative western blot of Bax, Bcl-2 and β-actin for the respective doses of RES in TRAMP-C1, TRAMP-C2 and TRAMP-C3 cells. **B**. Expression of Bcl-2 relative to the control. **C**. Expression of Bax relative to the control. Results are representative of three independent experiments (* indicates p<0.05 and # indicates p>0.05).

Western blots were performed to investigate the effect of RES on the expression of Bax and Bcl2 proteins. It was found that RES treatment modulated the expression of Bax and Bcl2 proteins in TRAMP-C1, TRAMP-C2 and TRAMP-C3 cells as compared to the control (Figure 3A). Further, densitometric analysis revealed that treatment with 50 μM or 100 μM of RES resulted in significantly high expression of Bax in TRAMP-C1 (*p<0.02), TRAMP-C2 (*p<0.001) and TRAMP-C3 (*p<0.001) cells when compared to the respective control (Figure 3C). In contrast to Bax, Bcl2 expression repressed significantly in TRAMP-C2 (*p<0.03) and TRAMP-C3 (*p<0.02) cells after 100 μM of RES treatment (Figure 3B). In addition, TRAMP-C1 (#p>0.05) cells did not show a significant difference in the expression of Bcl2 protein in comparison to the control (Figure 3B).

### RES induces caspase-independent cell killing

**Figure 4:**
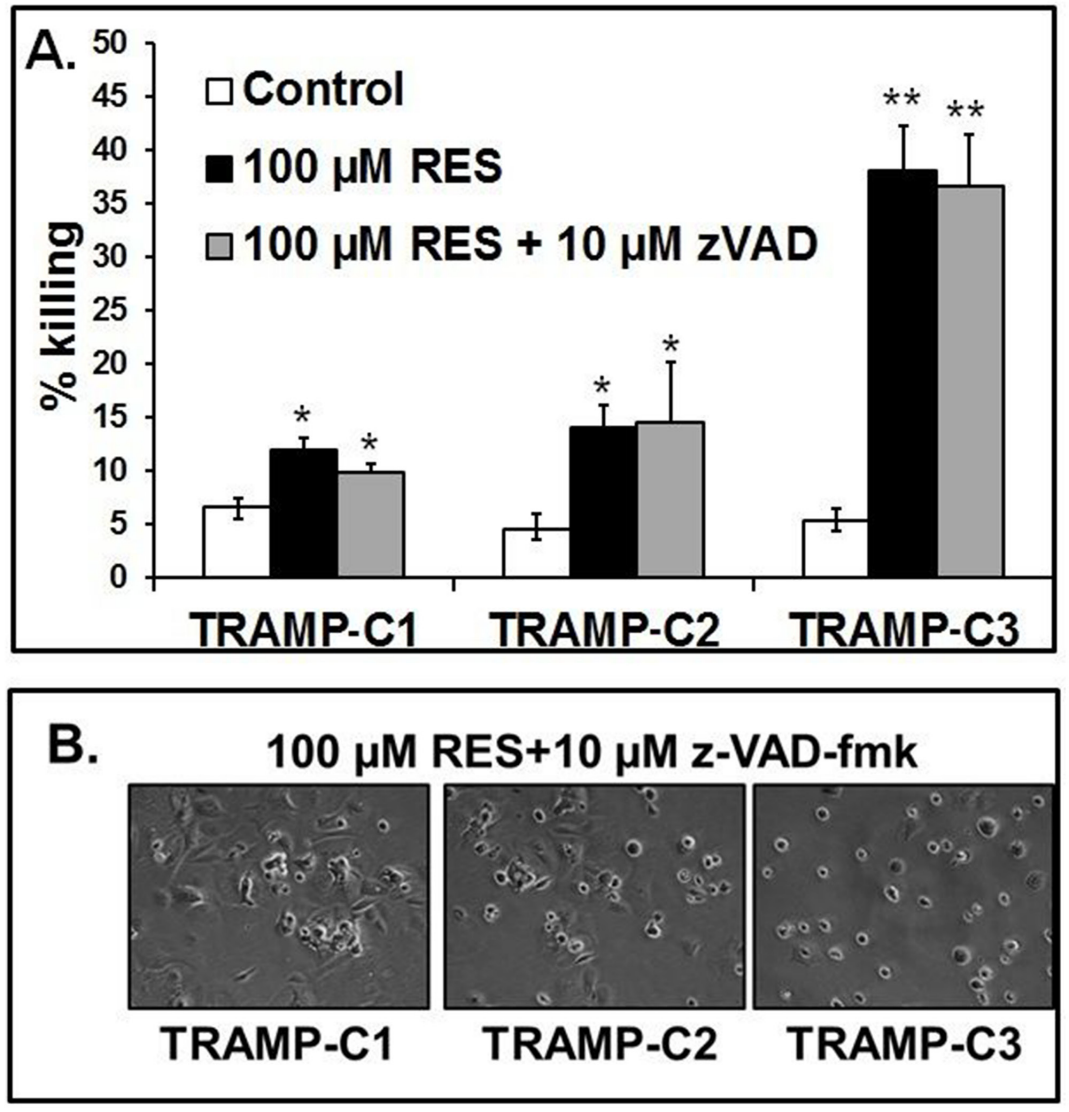
RES causes caspase-independent cell killing Cells were treated with 100 μM of RES with or without 10 μM of z-VAD-fmk (a broad-spectrum caspase-3 inhibitor) and incubated for 16 hours. Thereafter, annexin V-PI staining was accomplished. **A**. The percent of cell killing by RES in the presence or absence of 10 μM of z-VAD-fmk. **B**. Morphological alterations in TRAMP cells caused by 100μM of RES treatment in the presence of z-VAD-fmk. Data indicated mean values of ± SEM (* indicates p<0.05 and ** indicates p<0.001).

To evaluate the role of caspase-3 in RES-mediated cell killing, caspase-3 activation was blocked with an inhibitor (z-VAD-fmk). RES treatment with and without z-VAD-fmk induced RES-mediated cell killing (Figure 4A). TRAMP-C3 cells exposed to 100 μM of RES with or without z-VAD-fmk showed significantly (**p<0.001) higher percentage of cell death as compared to TRAMP-C1 (*p<0.05) and TRAMP-C2 (*p<0.05) cells (Figure 4A). However, there was no significant difference in TRAMP cells treated with either RES or with RES plus z-VAD-fmk (Figure 4A). There were corresponding results when cells were analyzed morphologically under a phase contrast microscope (Figure 4B: resultant morphology of TRAMP cell after 100μM of RES+z-VAD-fmk treatment). In addition, treatment with Nec-1 (an inhibitor of necroptosis) did not change RES-mediated cell killing in the presence or in the absence of z-VAD-fmk (Supplementary Figure 2). Thus, in TRAMP-C1, TRAMP-C2, and TRAMP-C3 cells, the caspase-3 inhibitor, z-VAD-fmk, and Nec-1, had negligible effects on RES-mediated apoptosis (Supplementary Figure 2A; TRAMP-C1, Supplementary Figure 2B; TRAMP-C2, and Supplementary Figure 2C; TRAMP-C3 cells).

### RES modulates the γ-H2A.X expression

**Figure 5:**
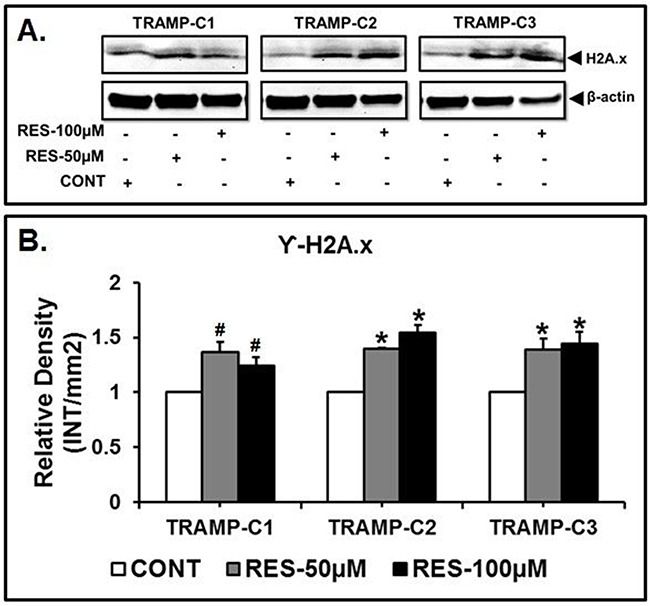
RES treatment induced DNA fragmentation in TRAMP cells TRAMP cells were incubated with 50 μM and 100 μM of RES to examine the expression of γ-H2A.X in TRAMP cells. The resulting western blots of γ-H2A.X showed that the expression of γ-H2A.X was found significantly higher in TRAMP-C2 and TRAMP-C3 cells when compared to the control. Furthermore, TRAMP-C1 cells, did not show a significant difference in γ-H2A.X expression when compared to the control (* indicates p<0.05 and # indicates p>0.05).

To test the effect of 50 and 100μM of RES treatment on the expression of γ-H2A.X in TRAMP cells. The expression of γ-H2A.X was examined using western blot analysis which demonstrated significantly (*p<0.05) higher expression in TRAMP-C2 and TRAMP-C3 cells when compared to the control (Figure 5A and 5B). However, in TRAMP-C1 cells, γ-H2A.X expression was not significant (#p>0.05) as compared to the control (Figure 5A and 5B). These findings suggest that RES treatment sensitizes DNA damage which further leads to apoptosis of TRAMP cells.

## DISCUSSION

**Figure 6:**
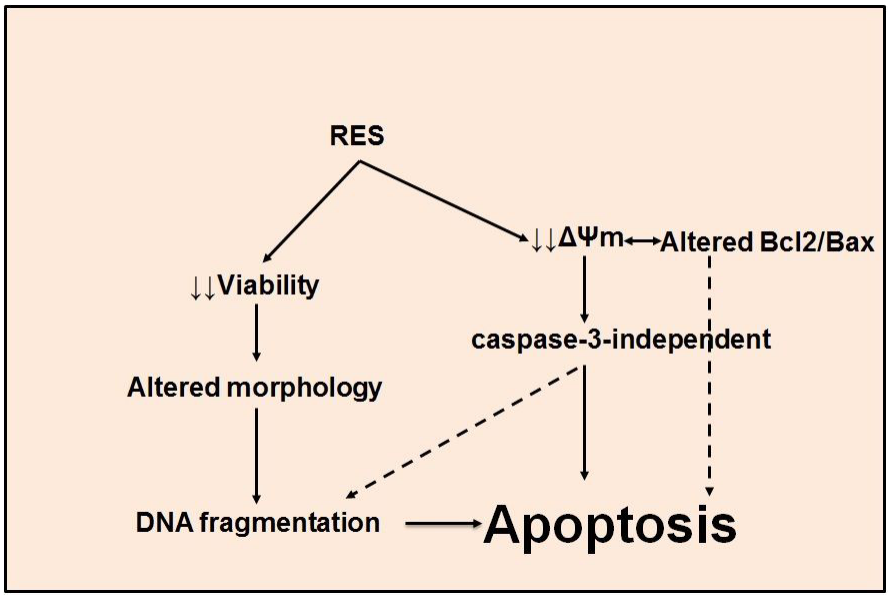
RES induces apoptosis in TRAMP cells A schematic representation of the mechanism of RES-mediated, caspase-independent apoptosis in TRAMP cells.

Resistance to anti-cancer therapies is facilitated through an array of mechanisms that vary across tumor types [63, 64]. In the present study, we demonstrated that RES modulates mitochondria-mediated, caspase-independent apoptosis in murine prostate cancer cells. These results reveal that dietary compounds such as RES may play a critical role in inducing mitochondria-dependent apoptotic pathway(s) in murine prostate cancer cells (Figure 6).

Dietary compounds can be used to target mitochondria-mediated, caspase-independent apoptosis. This pathway is characterized by changes in Δψm and by maintenance of an optimal ratio of Bcl2/Bax [65]. A high Δψm and a high Bcl2/Bax ratio are believed to promote cell proliferation and enhanced cell survival, thereby contributing to cancer progression. Dietary compounds such as RES inhibit cell growth in several types of human cancers, including prostate cancers [8, 66–70]. Consistent with results of previous studies, our data demonstrate that RES treatment results in enhanced cell killing in a time and dose-dependent manner. As cell viability decreases, altered cell morphology, a characteristic of apoptosis, increases [71]. RES induces cellular morphological changes similar to those caused by other anti-cancer drugs [40–45, 61, 62, 72]. To confirm our preliminary results relating to viability, we determined the effect of RES on the expression of Bcl2 and Bax proteins, Δψm, caspase-3 activity, and DNA fragmentation. TRAMP-C3 and TRAMP-C2 cells exhibited significant difference in Bax expression as compared to control at 50 μM and 100 μM of RES; however, TRAMP-C1 showed significant difference at 100 μM of RES, not at 50 μM when compared to the control (Figure 3C). In contrast to Bax, Bcl2 expression in TRAMP-C1, TRAMP-C2 and TRAMP-C3 was observed altered as compared to control at both 50 μM and 100 μM of RES (Figure 3B). Additionally, Bcl2 was found significantly repressed at 50 μM and 100 μM of RES in TRAMP-C2 and TRAMP-C3 when compared to control (Figure 3B). Furthermore, RES treatment also caused disrupted Δψm, which is associated with apoptosis [73]. Most TRAMP-C3 cells showed decreased Δψm relative to TRAMP-C1 and TRAMP-C2 cells. Thus, RES treatment induced killing of TRAMP cells in a caspase-3-independent manner, for the caspase-3 inhibitor (z-VAD-fmk), failed to prevent RES-mediated cell killing. Therefore, apoptosis of TRAMP cells induced by RES apparently acts through the mitochondrial mediated, caspase-independent pathway (Figure 6).

Changes in Δψm are evident in mitochondria-mediated, caspase-independent apoptosis [74, 75]. As shown in the present effort, RES treatment decreases Δψm in TRAMP-C3 cells compared to TRAMP-C1 and TRAMP-C2 cells. However, RES induces apoptosis of colon cancer cells independently of the tumor suppressor p53 via epithelial differentiation and mitochondrial membrane collapse [76, 77]. The present data correspond with these findings, which suggest that RES treatment induces a collapse of Δψm [76, 77].

Proteins of the Bcl2 family, particularly Bcl2 and Bax, are involved in mitochondria-mediated apoptotic pathways [65]. After treatment of HCT-116 colon carcinoma cells with RES, Bax is involved in alteration of mitochondrial membrane permeability [78–80]. However, by up-regulation of Bcl2 and inhibition of p53 and Bax, RES reverses cadmium chloride-induced testicular damage and subfertility [79]. In bladder cancer cells, RES also induces apoptosis by down-regulating the expression of Bcl2 proteins [81, 82]. However, the current results show that RES modulates the expression of Bax and Bcl2 in TRAMP-C1, TRAMP-C2, and TRAMP-C3 cells compared to the control cells; however, in TRAMP-C1 cells Bax at 50 μM of RES and Bcl2 at 50 μM and 100 μM of RES were found insignificant. These findings corroborated with other reports [65, 81, 82].

RES decreased cell viability and Δψm, and modulated the expression of Bax and Bcl2 proteins in TRAMP-C1, TRAMP-C2, and TRAMP-C3 cells. In an investigation of the involvement of caspase-3 in RES-mediated cell killing and DNA fragmentation, we found that RES treatment with or without the caspase-3 inhibitor, z-VAD-fmk, resulted in similar cell killing. This result was supported by morphological examination of the cells after DePsipher staining. The mechanism of action, however, is not yet defined. RES induces apoptosis by depolarizing the mitochondrial membranes in a caspase-independent manner [73] [48, 83, 84]. Nevertheless, in U937 cells, overexpression of Bcl2 attenuates RES-mediated apoptosis by blocking caspase-3 activation [53]. Moreover, RES induces caspase-dependent and -independent apoptosis in various cancer cells [85–87]. In colon cancer cells, RES induces caspase-2 activation that subsequently triggers Bax-Bak-dependent and -independent cell death [79]. In human lung adenocarcinoma cells, RES stimulates mitochondria-mediated and caspase-dependent cell death [88]. Conversely, in primary mouse fibroblasts, RES exhibits a cytoprotective effect by acting against caspase-3 [89]. The present results show that, for TRAMP cells, the caspase-3 inhibitor, z-VAD-fmk, and Nec-1, which blocks necroptosis, had a negligible effect on RES-mediated cell killing. Thus, in these cells, RES induces caspase-independent apoptosis.

The present results show that RES treatment to TRAMP cells caused significant cleavage of genomic DNA, which was accomplished by the expression of γ-H2A.X, an evolutionary conserved variant of histone H2A, has been identified as one of the key histones to undergo various post-translational modification in response to double stranded DNA breaks [90, 91]. DNA damage caused by radiation, UV light, or anti-cancer agents results in phosphorylation of Histone γ-H2A.X at ser-139 by PI3K-like kinases, including ATM, ATR, and DNA-PK [92–94]. The DNA damage response during DNA fragmentation is required for DNA-damage response proteins including DNA-PK that phosphorylates γ-H2A.X [95, 96]. Phosphorylation of γ-H2A.X at Tyr142 inhibits the recruitment of DNA repair proteins and promotes binding of pro-apoptotic factors such as JNK1 [97, 98]. Thus, γ-H2A.X expression was significantly higher in TRAMP-C2 and TRAMP-C3 cells after 50 and 100μM of RES treatment as compared to the control. However, in TRAMP-C1 cells, γ-H2A.X expression was not significant as compared to the control. These findings are consistent with the previous results, which show RES-mediated caspase-independent cell killing. Furthermore, previous studies have demonstrated that RES induces apoptosis and DNA fragmentation in several types of cancer cells [99–102]. These properties of RES suggest that it could be used as a therapeutic agent to treat prostate cancer.

Our results demonstrate that, for TRAMP-C1, TRAMP-C2, and TRAMP-C3 cells, RES increases cell killing in a dose- and time-dependent manner, induces morphological alterations, and triggers apoptosis. In these cells, RES causes a disrupted Δψm that leads to modulated expression of Bax and Bcl2 proteins. Additionally, caspase-3 is not involved in RES-mediated cell killing, showing that, in TRAMP cells, RES induces caspase-independent apoptosis. In these cells, RES treatment contributed to DNA fragmentation which enhanced γ-H2A.x expression in treated TRAMP-C2 and TRAMP-C3 cells, but not in TRAMP-C1 when compared to the control, indicating the sign of DNA damage after RES treatment [92]. Therefore, RES may be a promising dietary compound for the treatment of prostate cancer. However, further investigations are necessary to uncover the underlying mechanisms.

## MATERIALS AND METHODS

### Cell lines and culture conditions

TRAMP-C1, TRAMP-C2, and TRAMP-C3 cells were obtained from American Type Culture Collection (www.ATCC.org) and were maintained at 37° C under 5% CO_2_ in Dulbecco's Modified Eagle Medium (DMEM) supplemented with 10% fetal bovine serum (FBS) (v/v), bovine insulin (0.005 mg/ml), dehydroisoandrosterone (10 nM), and antibiotics/antimycotics (1%).

### Cell killing assay

To assess percentages of cell death, 70-80% confluent cells were harvested by trypsinization, counted, and seeded (10^5^) in 24-well plates in 1 ml of culture medium. Cells were treated with 50 μM or 100 μM of RES and were analyzed by flow cytometry at 0, 2, 4, 8, 12, and 16 hours. They were stained with annexin V-propidium iodide (PI) as directed by manufacturer. Briefly, into each tube, annexin V (5 μl of 600 μg/ml) and PI (5 μl of 30 μg/ml) were added, and tubes were then incubated for 15 min at 4°C. Cells were washed with Dulbecco's phosphate buffered saline (DPBS) and centrifuged. For flow cytometric analysis, the cells were suspended in 200-500 μl of annexin V binding buffer. Data were acquired by use of a 13-color flow cytometer (Novocyte, Acea Biosciences, San Diego, CA). PI^+^, Annexin V^+^, and Annexin V^+^ PI^+^ cells were counted as dead populations; PI/Annexin V FITC^−^ cells were counted as live cells. All experiments were performed in triplicate.

### Assessment of cell morphology

Cells were exposed to 50 μM or 100 μM of RES for 16 hours. Media were removed, and after media removal, cells were washed with DPBS and then suspended in 50 μl of DPBS. To assess morphological changes, cells were observed under a phase-contrast microscope (Life Tech, Grand Island, NY).

### Assessment of mitochondrial membrane potential

To examine Δψm, cells in DMEM supplemented with 10% FBS were exposed to RES (50 μM or 100 μM) for 16 hours. After incubation, cells were harvested, washed, stained with the mitochondria-specific dye, DePsipher (Trevigen, Gaithersburg, MD) and flow analyzed as suggested by the manufacturer. DePsipher, a cationic dye (5,5′6,6′-tetrachloro-1,1′,3,3′-tetraethylbenzimidazolylcarbocyanine iodide) stains both healthy cells and cells with disrupted Δψm. The dye enters into healthy mitochondria and, in its multimeric form, fluoresces red. However, in apoptotic cells, the dye remains in the cytoplasm and fluoresces green while in its monomeric form. Thus, cells with disrupted mitochondria can be differentiated from healthy cells. Cells were observed under a fluorescent microscope for morphology (Nikon ECLIPSE Ti, Melville, NY).

### Western blotting

Total protein was extracted from TRAMP cells (10^6^) treated with RES (50 μM or 100 μM) by use of 2x radioimmunoprecipitation assay buffer (RIPA). The concentrations of total proteins in lysates were estimated according to Bradford et al. [57], using bovine serum albumin as the standard. Estimated concentration of total proteins (30-40 μg/well) was run on 12% SDS-PAGE. Protein complexes were then transferred to nitrocellulose membranes, which were blocked in 5% skimmed milk and then incubated overnight with mouse anti-Bax (1:500) or anti-Bcl2 (1:100) monoclonal antibodies (Trevigen, Gaithersburg, MD and Thermo Scientific, NY, respectively). After repeated washing, the membranes were treated with a goat anti-mouse secondary antibody (1:1000) for 1 hour at room temperature. Proteins on the membranes were detected using an ECL-liquid substrate system (BioRad, Hercules CA). As an internal control, β-actin antibody (Grand Island, NY) was used to measure β-actin.

Next, in a separate experiment, estimated concentration of total proteins (20-40μg/well) were electrophoresed on 10% SDS-polyacrylamide gels. Protein complexes were transferred on nitrocellulose membranes (cat#162-0112: BioRad, CA, USA) and incubated with rabbit anti-γ-H2A.X polyclonal antibody (cat#2595: Cell signaling technology, MA, USA). Membranes were washed and incubated with goat anti-rabbit secondary antibody (cat#31460: Thermoscientific, NY, USA). Protein blots were visualized using super signal west femto ECL western blotting detection system (cat#34095: Thermoscientific, NY, USA), equal amount of proteins loading were tested by reprobing with anti-b-actin antibody (cat#3700S: Cell signaling technology, MA, USA).

### Assessment of caspase-independent cell death

To determine the role of caspases in RES-mediated cell death, cells were exposed to RES (100 μM) with or without 10 μM of z-VAD-fmk (a broad-spectrum caspase inhibitor) (Thermo Fisher Scientific, Grand Island, NY) for 16 hours. After incubation, caspase-independent cell death was determined. To accomplish this, cells were stained with annexin V (5 μl of 600 μg/ml) and PI (5 μl of 30 μg/ml) for 15 min at 4°C. Cells were washed with DPBS and suspended in 200-500 μl of annexin V buffer for flow cytometric analysis. Data were acquired by use of a 13-color flow cytometer (Novocyte, Acea Biosciences, San Diego, CA).

### Statistical analysis

Statistical significance of data was determined using Student's *t* test to determine the *p* value. For comparison of differences among the groups, single factor or multifactor one-way analysis of variance (ANOVA) followed by post hoc Bonferroni and Tukey test was used. Data were considered statistically significant at value p<0.05.

## SUPPLEMENTARY MATERIALS FIGURES


