# Supplementary material for: Resveratrol induces mitochondria-mediated, caspase-independent apoptosis in murine prostate cancer cells
Source: Oncotarget. 2017 Feb 1;8(13):20895–908. doi: 10.18632/oncotarget.14947 (PMC5400554; doi:10.18632/oncotarget.14947)
Supplement: Supplementary file 1 [file oncotarget-08-20895-s001.pdf]

# Resveratrol induces mitochondria-mediated, caspase-independent apoptosis in murine prostate cancer cells

## SUPPLEMENTARY FIGURES

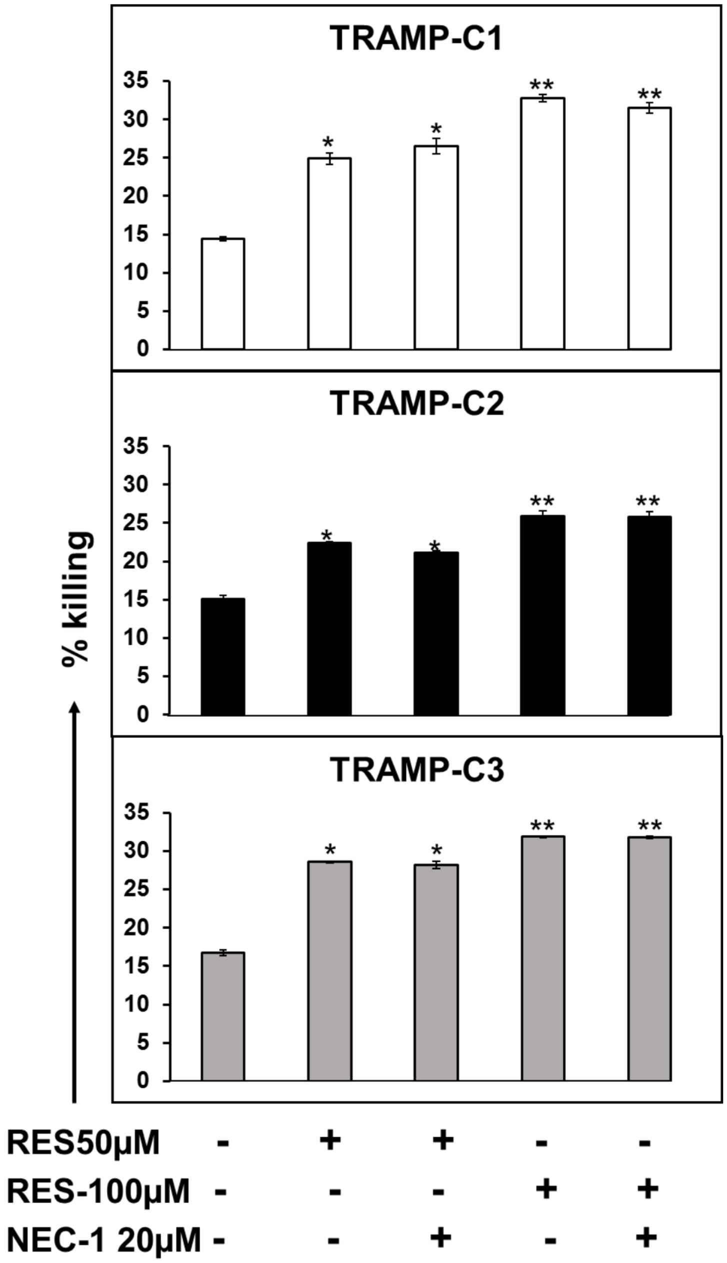


**Supplementary Figure 1: RES kills tumor cells.** No significant difference was evident when cells were incubated with RES (50 or 100 µM) in the presence or absence of Nec-1. The data shown are from three independent experiments performed in triplicates. **A.** TRAMP-C1 cells, **B.** TRAMP-C2 cells, and **C.** TRAMP-C3 cells respectively. Results represented as mean values of ±SEM (*p<0.05 and **p<0.01; control vs. treatment).

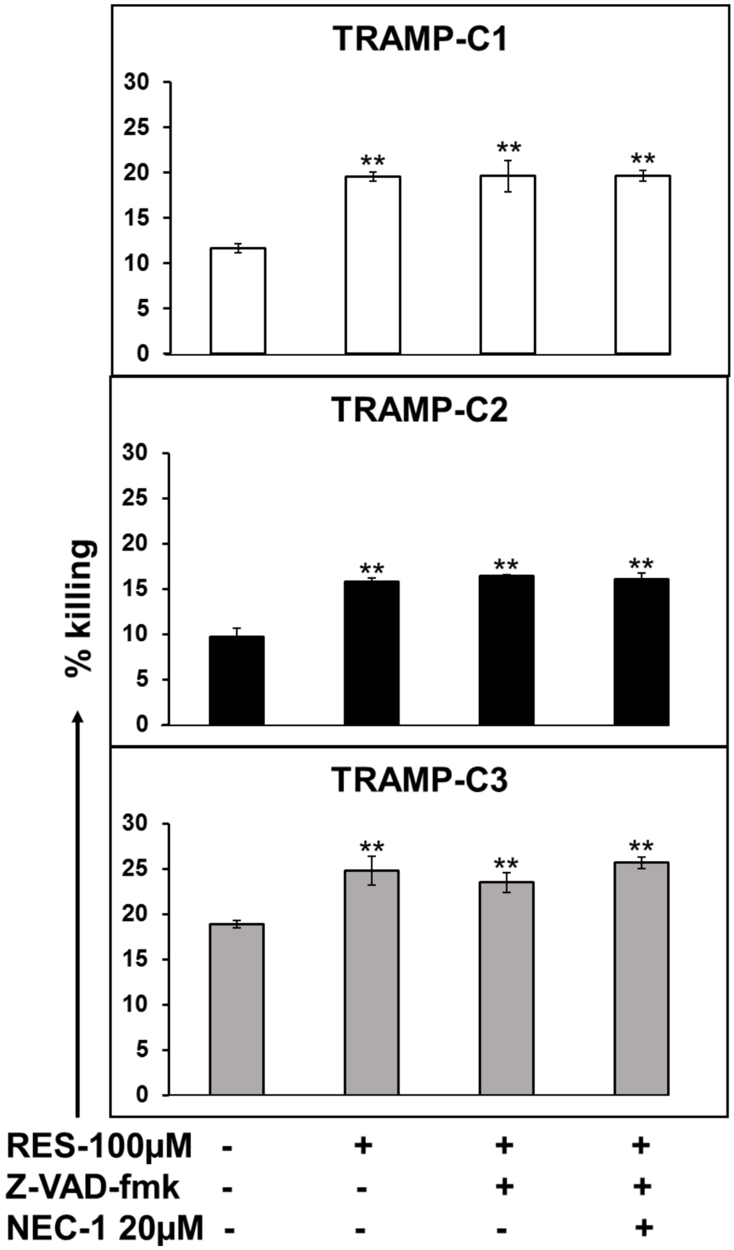


**Supplementary Figure 2: RES induces caspase-independent cell killing.** No significant difference was found when cells were exposed to 100µM of RES with or without z-VAD-fmk and Nec-1. Thus, RES kills tumor in a caspase-independent manner. The data are from three independent experiments performed in triplicate. Figure **A.** TRAMP-C1 cells, **B.** TRAMP-C2 cells, and **C.** TRAMP-C3 cells respectively. Data demonstrated mean values of ±SEM (**$p<0.01$; control vs. treatment).
